# Prognostic Role of the Circulating Tumor Cells Detected by Cytological Methods in Gastric Cancer: A Meta-Analysis

**DOI:** 10.1155/2016/2765464

**Published:** 2016-10-24

**Authors:** Kun Zou, Shuailong Yang, Liang Zheng, Shuyi Wang, Bin Xiong

**Affiliations:** Department of Oncology, Zhongnan Hospital of Wuhan University, Hubei Key of Laboratory of Tumor Biological Behaviors & Hubei Cancer Clinical Study Center, Wuhan 430071, China

## Abstract

*Objective*. We performed a meta-analysis of available studies to assess the prognostic value of circulating tumor cells detected by cytological methods for patients with gastric cancer.* Methods*. Two authors systematically searched the studies independently with key words in PubMed, MEDLINE, EMBASE, Science Citation Index Expanded, and Cochrane Library (from inception to April 2016). The estimated hazard ratio, risk ratio, odds ratio, and their 95% confidence intervals were set as effect measures. All analyses were performed by STATA 12.0.* Results*. Sixteen studies were included in this meta-analysis. CTCs-high status was significantly associated with poor overall survival (HR = 2.23, 95% CI: 1.86–2.66) and progression-free survival (HR = 2.02, 95% CI: 1.36–2.99). CTCs-high status was also associated with depth of infiltration (OR = 2.07, 95% CI: 1.16–3.70), regional lymph nodes metastasis (OR = 1.85, 95% CI: 1.26–2.71), and distant metastasis (OR = 2.83, 95% CI: 1.77–4.52). For unresectable gastric cancer patients, CTCs-high status was significantly associated with poor overall survival, progression-free survival, and disease control rate before and during chemotherapy group.* Conclusions*. Our meta-analysis has evidenced the significant prognostic value of CTCs detected for both PFS and OS in gastric cancer patients. For patients treated with chemotherapy alone, we proved that CTCs detected by cytological method showed a significant prognostic value and poor response to chemotherapy.

## 1. Introduction

Gastric cancer is the fifth most common malignant neoplasm and the third leading cause of death from cancer [1]. Most patients relapse after a prior curative surgical approach [2]. So far, pathological stage, histological type, lymphatic vessels, and vascular infiltration were widely used as prognostic factors of patients with gastric cancer. But all of them had limitations, and new and better predictors of survival of patients with gastric cancer were needed. Since the circulating tumor cells (CTCs) were discovered in peripheral blood of the patient with cancer in 1896, CTCs have been used in many aspects of cancer management, such as monitoring tumor recurrence and treatment efficacy, determining drug-selection strategies, and predicting the survival of cancer patients [3]. Recently, meta-analyses of CTCs' prognostic value have been confirmed in patients with lung cancer [4], breast cancer [5], and colorectal cancer [6].

Due to the low concentration in peripheral blood and the limited technology on CTCs detection, general inspection methods find it difficult to detect the rare cells and there is no widely accepted method in detecting the CTCs in gastric cancer. Currently, the major techniques used to identify CTCs can be divided into two aspects, the cytological methods (such as CellSearch, immunocytochemistry, flow cytometry, and immune-magnetic and fluorescence-activated cell sorter) and the molecular methods (mainly the PCR) [3]. Although meta-analyses have shown that the presence of CTCs in peripheral blood of patients with gastric cancer was associated with poor prognosis and clinical characteristics [7–9], most studies involved in these meta-analyses used the molecular methods and the prognostic value of CTCs detected by cytological methods remains controversial. The pooled HR on OS from two meta-analyses showed different results for CTCs positive patients when detected by cytological methods (HR = 2.00, 95% CI: 0.1.28–3.13 [7]; HR = 1.67, 95% CI: 0.57–4.92 [9], resp.) and the number of the involved studies was very little (two and three, resp.). So there were limitations in them. Besides, new studies using the cytological methods have been reported recently. Therefore, it is necessary to carry out a new meta-analysis on the prognostic role of CTCs in patients with gastric cancer.

With the controversies existing in the prognostic role of CTCs for gastric cancer, here, we conducted the meta-analysis of published literature on this topic to summarize the evidence of the clinical and prognostic role of CTCs detected by cytological methods in gastric cancer patients.

## 2. Methods

### 2.1. Search Strategy

Two authors systematically searched the studies independently with key words “gastric cancer”, “circulating tumor cells”, “prognosis”, and “peripheral blood” in PubMed, MEDLINE, EMBASE, Science Citation Index Expanded, and Cochrane Library (from inception to April 2016). An additional search through Google Scholar and the clinical trial registration website was conducted to obtain information identifying other potentially relevant publications. Discrepancies were resolved by the third author. In order to ensure the integrity of the retrieval, we also conducted a manual search.

### 2.2. Inclusion and Exclusion Criteria

The inclusion criteria were as follows: (1) studies using any kind of cytological methods to evaluate the association between the circulating tumor cells and overall survival (OS), progression-free survival (PFS), or clinic-pathological characteristics of gastric cancer; (2) sufficient data to calculate a hazard ratio (HR), risk ratio (RR), or odds ratio (OR) and 95% confidence interval (95% CI) being available; (3) at least 20 patients being involved in the studies; (4) samples being collected from the peripheral blood.

The exclusion criteria were as follows: (1) samples coming from lymph nodes, the peritoneal cavity, or bone marrow; (2) studies based on overlapping patients; (3) meta-analysis, review, single test, case report, conference reports and experiments, reporting the expert experience; (4) outcome being unclear or the apparent paradox existence; (5) unattainability of enough data after contacting the original author or magazine.

### 2.3. Data Extraction

Data retrieved from the studies included the first author's name, year of publication, number of patients, detection method, CTCs-high number, country (or area) of patients, sampling times (before the initiation of surgery and chemotherapy [“baseline”] or after the initiation of chemotherapy [“during chemotherapy”]), population of the patients (resectable or unresectable) and prognostic value (OS and PFS), disease control rate (DCR) to chemotherapy, tumor clinic-pathological characteristics, and hazard ratio (HR). For studies with multiple arms (i.e., resectable and unresectable groups), each of the subgroups was considered an independent data set. For studies with multiple time points (i.e., baseline and during chemotherapy), we used data from “baseline” samples prior to the data from “during chemotherapy” samples because those data were usually dependent. If the HR and its 95% CI were not reported directly in the original study, these values were calculated from available reported data using software designed by Tierney et al. [26]. All data was extracted independently by two investigators. The discrepancy between the reviewers was finally achieved through consultation. We used the Newcastle-Ottawa scale (NOS) [27] to assess the quality of cohort studies which was recommended by the Cochrane Library for observational studies, where a score of 5–9 means high quality and a score of 1–4 means low quality. This article follows the QUORUM and the Cochrane Collaboration guidelines (http://www.cochrane.de) for reporting meta-analysis (PRISMA statement) [28].

### 2.4. Statistical Analysis

All analyses of the data in our meta-analysis were performed using the STATA 12.0 package (StataCorp, College Station, TX, USA). The estimated HR was used to evaluate the prognostic effect (PFS and OS) as demonstrated by Parmar et al. [29] and HR > 1 reflected more deaths or progression in the CTCs-high arm. Besides, the estimated odds ratio (OR) was used to summarize the association between CTCs detection and gastric tumor clinic-pathological characteristics, and the estimated risk ratio (RR) was used to evaluate the efficacy of chemotherapy (DCR). All statistical values (pooled HR, RR, and OR) were combined with a 95% CI and the *P* value threshold was set at 0.05. Heterogeneity was assessed by *I* ^2^ inconsistency test and *χ* ^2^ based Cochran's *Q* statistic test [30] in which *I* ^2^ > 50% or *P* < 0.1 indicated significant heterogeneity. When *I* ^2^ < 50% and *P* > 0.1, the fixed effect model was used, or the random effects model was used conversely [31]. Publication bias was detected by Begg's test and Egger's test [32]. *P* < 0.05 was considered of significant publication bias. Furthermore, subgroup analyses were made according to the difference of the data retrieved such as country, methodology, population of the patients, CTC-high number, and quality of the studies. Subgroup analyses were performed only when there were two or more studies included in the subgroups. And in order to explore the potential sources of heterogeneity, we also did univariate metaregression analyses (random effects) on the same factors.

## 3. Result

### 3.1. Baseline Study Characteristics

**Figure 1:**
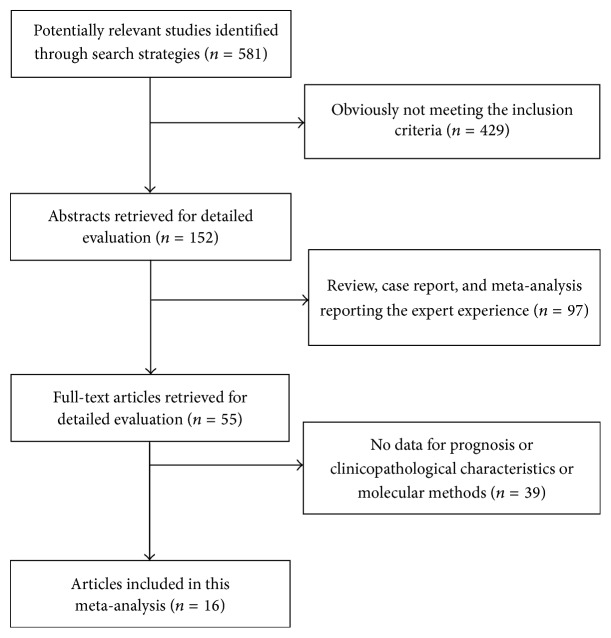
Selection of the included studies.

According to the mentioned retrieval method, 581 potentially relevant studies were assessed. Detailed steps of the search were shown (Figure 1). After the selection procedure, 16 cohort studies with a total of 1110 gastric cancer patients were included [10–25]. The basic characteristics and the quality assessment of these studies were shown in Table 1. These studies were from seven countries (China, Japan, Korea, Poland, USA, UK, and Netherlands) and were published between 2007 and 2016. Four of the retrieved studies only provided the association between the CTCs and clinic-pathological characteristics.

12 studies mentioned the prognostic significance of the CTCs; and seven of the studies used the CellSearch method, eight provided the prognostic information of the unresectable gastric cancer patients treated with chemotherapy alone, and one had two independent data sets with multiple arms. Besides, three of the studies had two data sets with multiple time points. As to the quality assessment shown in Table 1, three of the 12 studies were of low quality and the other 9 studies were of high quality.

#### 3.1.1. The Prognostic Effect (OS and PFS) of CTCs Detection

**Figure 2:**
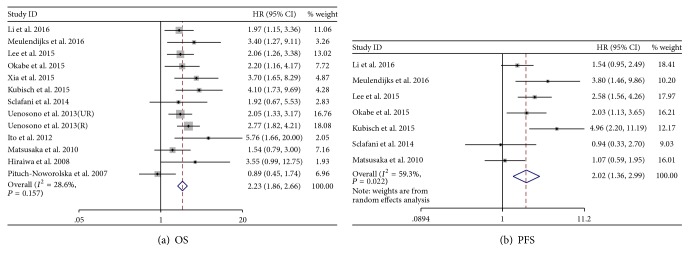
Hazard ratio (HR) for overall survival (OS) and progression-free survival (PFS) of the included studies.

All 12 studies were available for the overall survival, and seven studies were available for the progression-free survival. There was no significant heterogeneity between these studies when pooling the HR on OS (*I* ^2^ = 28.6%, *P* = 0.157) and a fixed model was used; the pooled HR for OS was 2.23 (95% CI: 1.86–2.66) (Figure 2(a)). However, the heterogeneity for PFS (*I* ^2^ = 59.3%, *P* = 0.022) was significant, the random effects model was used, and the pooled HR was 2.02 (95% CI: 1.36–2.99) (Figure 2(b)). The pooled results showed that CTCs-high status detected by cytological methods was a significant prognostic factor for gastric cancer patients, and there were more deaths or progression in the CTCs-high arm than in the CTCs-low arm.

Furthermore, we stratified the included studies based on variables (such as country, population, methodology, CTCs-high number, and quality); the results were shown in Table 2. The results showed a significant prognostic effect for OS and PFS and demonstrated a higher risk of deaths or progression in the CTCs-high arm than in the CTCs-low arm for all subgroups. For PFS, the heterogeneity dropped to insignificant level when studies were stratified by methodology (*I* ^2^ = 39.9%, *P* = 0.155; and *I* ^2^ = 0.0%, *P* = 0.667, resp.). For OS, heterogeneity was eliminated in subgroups by exclusion of studies coming from non-East Asia countries, resectable patients, or non-CellSearch methods.

#### 3.1.2. OS, PFS, and DCR with CTCs Detection in Unresectable Patients

**Figure 3:**
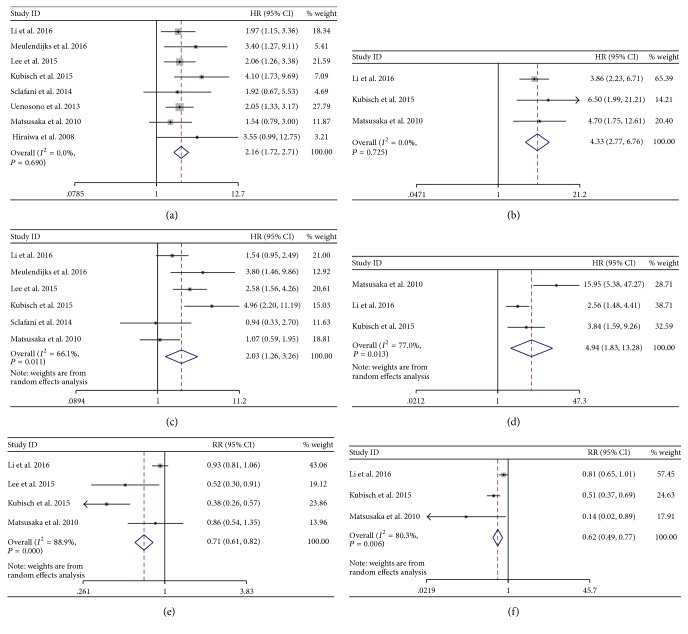
Hazard ratio (HR) for OS at baseline (a), OS in during chemotherapy (b), PFS at baseline (c), PFS in during chemotherapy (d), risk ratio (RR) for DCR at baseline (e), and DCR in during chemotherapy (f). OS: overall survival; PFS: progression-free survival; DCR: disease control rate.

Eight of the involved studies were designed for patients with unresectable or recurrent gastric cancer patients. As shown in Figure 3, eight data sets from baseline (before chemotherapy) samples of these studies were available for OS; the pooled analysis showed a prognostic effect of CTCs-high status (HR = 2.16, 95% CI: 1.72–2.71), with no significant heterogeneity between the studies (*I* ^2^ = 0.0%, *P* = 0.690) (Figure 3(a)). Six data sets with an significant heterogeneity (*I* ^2^ = 66.1%, *P* = 0.011) were available for PFS. The pooled HR for PFS was 2.03 (95% CI: 1.26–3.26) (Figure 3(c)). For the disease control rate (DCR), 4 studies were available. The pooled RR was 0.71 (95% CI: 0.61–0.82) With an significant heterogeneity (*I* ^2^ = 88.9%, *P* < 0.001) (Figure 3(e)). These results showed a poor prognosis and response to chemotherapy in the unresectable gastric cancer patients with CTCs-high status detected at baseline.

Besides, three studies also reported the prognostic value and the DCR for the CTCs-high status detected during chemotherapy. We pooled these data separately, and the results were shown in Figure 3. A poor prognosis and response to chemotherapy were found in CTCs-high status arm (OS: HR = 4.33, 95% CI [2.77–6.76]; PFS: HR = 4.94, 95% CI [1.83–13.28]; DCR: RR = 0.62, 95% CI [0.49–0.77]) (Figures 3(b), 3(d), and 3(f)).

#### 3.1.3. Correlation between Detection of CTCs and Clinic-Pathological Characteristics

**Figure 4:**
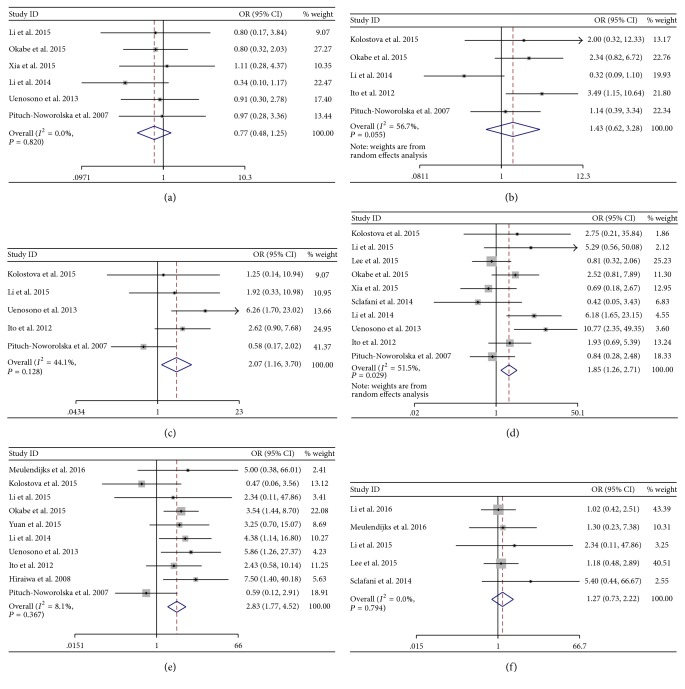
Odds ratio (OR) for sexuality (a), TNM stage (b) (III/IV versus I/II), depth of infiltration (c) (T3/4 versus T1/2), RLNs metastasis (d), distant metastasis (e), and peritoneum metastasis (f) associated with CTCs-high status. RLNs: regional lymph nodes.

We extracted clinic-pathological characteristics from the included studies. The potential correlation between detection of CTCs and clinical variables was investigated and showed in Figure 4, when the clinical variables were mentioned at least in 5 studies. The pooled odds ratio demonstrated that the incidence of CTCs was significantly different between the T3/T4 and T1/T2 groups (OR = 2.07, 95% CI: 1.16–3.70, *n* = 5) (Figure 4(c)), region lymph node metastasis positive and negative groups (OR = 1.85, 95% CI: 1.26–2.71, *n* = 10) (Figure 4(d)), or distant metastasis positive and negative groups (OR = 2.83, 95% CI: 1.77–4.52, *n* = 10) (Figure 4(e)). However, the pooled OR showed no significant difference between female and male, III/IV and I/II, or peritoneum metastasis positive and negative groups (Figures 4(a), 4(b), and 4(f)).

#### 3.1.4. Evaluation of Heterogeneity and Publication Bias

To explore the potential sources of heterogeneity, we conducted a meta-regression that considered the covariates of country, population, methodology, CTCs-high number, and quality for data from baseline samples. The results were shown in Table 3. In a univariate analysis, methodology showed a borderline explanatory variable that influenced the heterogeneity of estimated HR for PFS (coefficient = 0.980, standard error = 0.387, and *P* = 0.053), and it explained 73.92% proportion of between-study variance. However, other covariates were not significantly correlated with the heterogeneity across studies on PFS. For OS, none of these covariates was significantly correlated with the heterogeneity across studies on OS; this was in accordance with the little heterogeneity (*I* ^2^ = 28.6%, *P* = 0.157) and may indicate the consistency between the involved studies.

For the data from during chemotherapy samples, we performed sensitivity analyses to explore the potential sources of heterogeneity and test whether the results were stable. And the results were showed in Table 4. Sensitivity analyses indicated that the study by Matsusaka et al. [19] was the main origin of the heterogeneity for PFS. After the exclusion of the study, the heterogeneity for PFS was removed. This may be due to the limited CTC-high patients of the study by Matsusaka et al. (only nine patients). And while we deleted any one of the studies from the overall pooled analysis each time, the pooled HR for OS and PFS still remained significant. This indicated that the pooled results were stable.

Publication bias was detected by Begg's test and Egger's test. *P* < 0.05 confirmed the existence of publication bias. No publication bias was shown in OS (Begg's *P* = 0.300, Egger's *P* = 0.311) and PFS (Begg's *P* > 0.999, Egger's *P* = 0.672).

## 4. Discussion

Gastric cancer is a very common disease with high rates of prevalence and mortality in the world [1]. Although great progress has been made in the treatment of gastric cancer, the five-year survival rate was still below 30% [2]. Recently, CTCs have been shown to have an important role in tumor metastasis, and their significant prognostic value has also been demonstrated in several cancers [4–6]. In this meta-analysis, we provided strong evidence that CTCs detected by cytological methods in peripheral blood were significantly associated with poor PFS and OS of gastric cancer patients, irrespective of the geographical, population, and detection methods and CTCs-high number differences.

The result of our meta-analysis solved the controversies from two independent meta-analyses [7, 9] and demonstrated the prognostic role of CTCs detected by cytological methods in gastric cancer. Cytological methods may avoid false positive results from nonneoplastic and contaminated samples which was frequent in molecular methods, and they were able to count the number of CTCs and recognize viable and functional CTCs [33], so they may provide us with a more accurate result by using the cytological methods. Besides, to our knowledge, this was the first meta-analysis that assessed the prognostic and predictive value of CTCs in unresectable gastric cancer patients treated with chemotherapy alone.

According to the results in our meta-analysis, CTCs detected by cytological method have shown an significant prognostic value and association with some of the clinic-pathological characteristics in gastric cancer patients. The pooled results showed more deaths or progression in the CTCs-high arm than in the CTCs-low arm, and this result was also found in all subgroups when we stratified the included studies based on variables (Table 2). These results demonstrated that CTCs-high status detected at baseline indicated poor prognosis in gastric cancer patients and these patients may need more aggressive treatment and frequently efficacy assessments.

Furthermore, a significant heterogeneity was found in PFS (*I* ^2^ = 59.3%, *P* = 0.022). To explore the potential sources of heterogeneity, we made subgroup analyses and found that the heterogeneity dropped to insignificant level when studies were stratified by methodology (*I* ^2^ = 39.9%, *P* = 0.155; and *I* ^2^ = 0.0%, *P* = 0.667, resp.). Then, in meta-regression, methodology also showed a borderline explanatory variable for the heterogeneity on PFS (coefficient = 0.980) and explained 73.92% proportion of between-study variance. So we finally confirmed methodology had positively contributed to heterogeneity on PFS. This may be explained by the multiple cytological methods. The approaches for CTC isolation/enrichment and techniques for CTC detection/identification used in those cytological methods were different; then, the specificity and reliability of its detection were also different. And as CTCs are generally thought to be quite heterogeneous in both phenotype and genotype, some specific CTCs may be ignored in some methods; for example, the CTCs that had undergone the epithelial-to-mesenchymal transition could hardly be detected by using CellSearch method and may be detected by using other methods. Besides, although heterogeneity was eliminated in subgroups by exclusion of studies coming from non-East Asia countries, resectable patients, or non-CellSearch methods, the meta-regression on OS showed none of the covariates was significantly correlated with the heterogeneity. Taking into account the little heterogeneity on OS (*I* ^2^ = 28.6%, *P* = 0.157), we confirmed the consistency on OS between the involved studies.

Moreover, we assessed correlation between detection of CTCs and clinic-pathological characteristics, and we found that CTCs were more frequent in T3/T4, lymph node metastasis positive, and distant metastasis positive gastric cancer patients. But no significant difference was found between female and male, III/IV and I/II, or peritoneum metastasis positive and negative groups. The negative results for peritoneum metastasis and TNM staging were mainly caused by the limited studies (only five studies involved). So more studies assessing correlation between CTCs and clinic-pathological characteristics were needed.

In this meta-analysis, we proved that CTCs-high status showed a significant prognostic value and poor response to chemotherapy in gastric cancer patients treated with chemotherapy alone. As the data used in the initial analysis was only from prechemotherapy samples, we also made an independent analysis for the data from during chemotherapy samples. And we found coincident result of poor prognosis and response to chemotherapy for CTCs-high status patients in prechemotherapy and during chemotherapy group. The CTCs-high status before/during chemotherapy can be used as a prediction marker for the prognosis and response to chemotherapy.

At the same time, we found a more conspicuous result for both prognostic effect and the response to chemotherapy (DCR) in during chemotherapy group than prechemotherapy group. The same result for prognostic effect was also observed in other studies [11, 15, 19]. As for the conspicuous result in during chemotherapy group, we thought it may be because CTCs can be eliminated by chemotherapeutic drugs through direct and indirect mechanisms, such as cytotoxic and antimetabolic effects. And the remaining CTCs after chemotherapy may be more aggressive than before, and it may be easy to form metastases or cause recurrence. According to the results in our meta-analysis, we thought that CTCs-high status exhibited during chemotherapy may indicate more resistance to the chemotherapy and be useful for monitoring therapeutic effect. Furthermore, for data from during chemotherapy, we confirmed that the pooled results were stable and the heterogeneity was caused by the study of Matsusaka et al., and this may be explained by the limited CTC-high patients number in Matsusaka et al. [19]. Generally, CTCs-high during chemotherapy could provide earlier opportunities for early intervention or for the adjustment of chemotherapy by changing the chemotherapeutic regimen, intensity, and/or period.

Besides, as shown in another meta-analysis for colorectal cancer [34] and one study [10] involved in our meta-analysis, fluctuations in CTC levels before and during chemotherapy were closely associated with the tumor response to chemotherapy and prognosis, and the decreases and increases of CTC number in posttherapy were associated with superior and inferior survival, respectively. But for the lack of the related data, we failed to analyze the fluctuations of CTCs. So, for the predicted role of the changes in CTCs, more high quality related articles were needed.

There are some limitations in our meta-analysis. Firstly, the meta-analysis used the pooled data which was extracted from heterogeneous studies, not original data from the individual patients. The total number of patients from the involved studies was relatively small. Large prospective studies for gastric cancer were absent in this meta-analysis. Secondly, multiple methods for CTCs detection were used in our meta-analysis, and the standard for CTCs-high status in our retrieved studies was also different. These may contribute to the heterogeneity and limit its uses. Besides, approaches based on cytological method have biologic specificity and can quantify the number of CTCs, but the efficiency and sensitivity for the detection of CTCs are relatively low compared with the molecular methods. At last, little studies were designed for the predicted role of the fluctuations in CTC, so this meta-analysis did not carry out the relative conclusion.

## 5. Conclusion

In conclusion, our meta-analysis has evidenced the significant prognostic value of CTCs detected for both PFS and OS in gastric cancer patients, and the detection of CTCs was associated with some clinic-pathological characteristics. For the patients treated with chemotherapy alone, we proved that CTCs detected by cytological method showed a significant prognostic value and poor response to chemotherapy. But, large prospective studies are needed to validate the prognostic values of the changes in CTC. Meanwhile, more high quality randomized controlled trials are needed to provide more information. And the same standardized detection platforms and number of the favorable CTCs are expected to normalize and reduce the inconsistencies across studies.

## Figures and Tables

**Table 1:** Baseline characteristics and quality assessment by the Newcastle-Ottawa scale of eligible studies.

Study	Number	Methodology	CTC-high number	Country	Population	Time points	End point	Stars
Li et al. 2016 [10]	136	CellSearch	≥3	China	UR	Baseline	OS/PFS	7
						During chemotherapy	OS/PFS	
Meulendijks et al. 2016 [11]	24	FACS-ICC	≥2	Netherlands	UR	Baseline	OS/PFS	5
Lee et al. 2015 [12]	100	CellSearch	≥5	Korea	UR	Baseline	OS/PFS	7
Okabe et al. 2015 [13]	136	CellSearch	≥1	Japan	R + UR	Baseline	OS/PFS	6
Xia et al. 2015 [14]	36	Flow cytometry	≥1	China	R	Baseline	OS	4
Kubisch et al. 2015 [15]	62	Immune-magnetic	≥1	USA	UR	Baseline	OS/PFS	7
						During chemotherapy	OS/PFS	7
Sclafani et al. 2014 [16]	22	CellSearch	≥2	UK	UR	Baseline	OS/PFS	4
Uenosono et al. 2013 [17]	251	CellSearch	≥1	Japan	UR	Baseline	OS	6
					R	Baseline	OS/RFS	
Ito et al. 2012 [18]	65	ICC	≥5	Japan	R	Baseline	OS	5
Matsusaka et al. 2010 [19]	52	CellSearch	≥4	Japan	UR	Baseline	OS/PFS	6
						During chemotherapy	OS/PFS	
Hiraiwa et al. 2008 [20]	27	CellSearch	≥2	Japan	UR	Baseline	OS	4
Pituch-Noworolska et al. 2007 [21]	57	FACS-ICC	≥1	Poland	R	Baseline	OS	7
Kolostova et al. 2015 [22]	22	MetaCell ICC	≥1	Poland	R + UR	Baseline	NR	3
Li et al. 2015 [23]	44	FACS	≥1	China	R	Baseline	NR	4
Li et al. 2014 [24]	45	FACS	≥1	China	R + UR	Baseline	NR	4
Yuan et al. 2015 [25]	31	FACS	≥1	China	R + UR	Baseline	NR	4

FACS: fluorescence-activated cell sorter; ICC: Immunocytochemistry; UR: unresectable; R: resectable; NR: unreported; Stars: 0–4 means low quality; 5–9 means high quality.

**Table 2:** Results of subgroup analyses on PFS and OS.

Variables	OS				PFS			
	HR [95% CI]	*n*	*I* ^2^ (%)	*P* ^*d*^	HR [95% CI]	*n*	*I* ^2^ (%)	*P* ^*d*^
Country								
East Asia	2.30 [1.89–2.80]	9	0.0	0.557	1.74 [1.21–2.50]	4	44.4	0.145
Non-East Asia	1.89 [1.23–2.90]	4	67.8	0.025	2.72 [1.04–7.13]	3	68.5	0.042
Population								
Resectable	2.38 [1.40–4.06]	5	66.3	0.018	—	1	—	—
Unresectable	2.16 [1.72–2.71]	8	0.0	0.690	2.03 [1.26–3.26]	6	66.1	0.011
Methodology								
CellSearch	2.17 [1.78–2.65]	8	0.0	0.870	1.69 [1.31–2.20]	5	39.9	0.155
Non-CellSearch	2.86 [1.39–5.90]	5	70.0	0.010	4.43 [2.39–8.23]	2	0.0	0.667
CTC-high *n* ≥ 3								
Yes	2.03 [1.49–2.77]	4	11.0	0.338	1.65 [1.02–2.68]	3	60.8	0.078
No	2.33 [1.87–2.90]	9	38.1	0.114	2.52 [1.32–4.79]	4	58.6	0.064
Quality								
High	2.15 [1.79–2.60]	10	38.2	0.103	2.17 [1.45–3.27]	6	60.9	0.026
Low	3.03 [1.71–5.37]	3	0.0	0.603	—	1	—	—
Overall	2.23 [1.86–2.66]	13	28.6	0.157	2.02 [1.36–2.99]	7	59.3	0.022

The superscript “*d*” refers to heterogeneity.

**Table 3:** Results of metaregression on OS and PFS.

Variables	OS				PFS			
	Coef.	Std. err.	*P*	Adj *R*-squared	Coef.	Std. err.	*P*	Adj *R*-squared
Country	−0.1778	0.2920	0.555	−9.31%	0.4922	0.4600	0.333	18.62%
Score	0.3353	0.3580	0.369	14.16%	−0.8383	0.7327	0.304	2.50%
Methodology	0.1810	0.2714	0.519	−79.51%	0.9800	0.3875	0.053	73.92%
Population	0.0445	0.2507	0.862	−70.30%	0.0001	0.6618	>0.999	−47.68%
CTC-high *n*	0.1265	0.2551	0.630	−47.27%	0.4266	0.4353	0.372	1.48%

Adj *R*-squared: proportion of between-study variance explained; *n*: number; Coef.: coefficient; Std. err.: standard error.

**Table 4:** Sensitivity analyses for data during chemotherapy samples.

Study omitted	OS				PS			
	HR	95% CI	*I* ^2^ (%)	*P* ^*d*^	HR	95% CI	*I* ^2^ (%)	*P* ^*d*^
Li et al. 2016 [10]	5.37	2.52–11.45	0.0	0.680	7.54	1.87–30.38	74.9	0.046
Kubisch et al. 2015 [15]	4.05	2.50–6.55	0.0	0.734	6.00	1.00–35.92	88.5	0.003
Matsusaka et al. 2010 [19]	4.24	2.57–6.99	0.0	0.435	2.86	1.80–4.55	0.0	0.441

Combined	4.33	2.77–6.76	0.0	0.725	4.94	1.83–13.28	77.0	0.013

The superscript “*d*” refers to heterogeneity.

## Abbreviations

CTCs: Circulating tumor cells
PFS: Progression-free survival
OS: Overall survival
DCR: Disease control rate
HR: Hazard ratio
RR: Risk ratio
OR: Odds ratio
95% CI: 95% confidence intervals.
